# Maximum and Minimum Phonatory Glottal Area before and after Treatment for Vocal Nodules

**DOI:** 10.3390/healthcare8030326

**Published:** 2020-09-07

**Authors:** Cheng-Ming Hsu, Ming-Yu Yang, Tuan-Jen Fang, Ching-Yuan Wu, Yao-Te Tsai, Geng-He Chang, Ming-Shao Tsai

**Affiliations:** 1 Department of Otolaryngology–Head and Neck Surgery, Chiayi Chang Gung Memorial Hospital, Chiayi 613, Taiwan; scm00031@gmail.com (C.-M.H.); yaote1215@gmail.com (Y.-T.T.); genghechang@gmail.com (G.-H.C.); 2 Faculty of Medicine, College of Medicine, Chang Gung University, Taoyuan 333, Taiwan; fang3109@cgmh.org.tw; 3 Department of Otolaryngology, Kaohsiung Chang Gung Memorial Hospital, Kaohsiung 833, Taiwan; yangmy@mail.cgu.edu.tw; 4 Graduate Institute of Clinical Medical Sciences, College of Medicine, Chang Gung University, Taoyuan 333, Taiwan; 5 Department of Otolaryngology–Head and Neck Surgery, Linkou Chang Gung Memorial Hospital, Taoyuan 333, Taiwan; 6 Department of Traditional Chinese Medicine, Chiayi Chang Gung Memorial Hospital, Chiayi 613, Taiwan; smbepigwu77@gmail.com; 7 School of Traditional Chinese Medicine, College of Medicine, Chang Gung University, Taoyuan 333, Taiwan

**Keywords:** vocal fold nodule, glottal area, phonosurgery, voice therapy

## Abstract

*Background*: Vocal fold nodules (VFNs) are a challenge for otolaryngologists. Glottal area (GA) waveform analysis is an examination method used for assessing vocal fold vibration and function. However, GA in patients with VFNs has rarely been studied. This study investigated the maximum and minimum GA in VFN patients using modern waveform analysis combining ImageJ software and videostroboscopy. *Methods*: This study enrolled 42 patients newly diagnosed with VFN, 15 of whom received voice therapy and 27 of whom underwent surgery. Acoustic parameters and maximum phonation time (MPT) were recorded, and patients completed the Chinese Voice Handicap Index-10 (VHI-C10) before and after treatment. After videostroboscopy examination, the maximum and minimum GAs were calculated using ImageJ software. The GAs of patients with VFNs before and after surgery or voice therapy were analyzed. *Results*: The MPTs of the patients before and after voice therapy or surgery did not change significantly. VHI-C10 scores decreased after voice therapy but the decrease was nonsignificant (14.0 ± 8.44 vs. 9.40 ± 10.24, *p* = 0.222); VHI-C10 scores were significantly decreased after surgery (22.53 ± 7.17 vs. 12.75 ± 9.84, *p* = 0.038). Voice therapy significantly increased the maximum GA (5.58 ± 2.41 vs. 8.65 ± 3.17, *p* = 0.012) and nonsignificantly decreased the minimum GA (0.60 ± 0.73 vs. 0.21 ± 0.46, *p* = 0.098). Surgery nonsignificantly increased the maximum GA (6.34 ± 3.82 vs. 8.73 ± 5.57, p = 0.118) and significantly decreased the minimum GA (0.30 ± 0.59 vs. 0.00 ± 0.00, *p* = 0.036). *Conclusion:* This study investigated the GA of patients with VFNs who received voice therapy or surgery. The findings indicated that voice therapy significantly increased maximum GA and surgery significantly decreased minimum GA. GA analysis could be applied to evaluate the efficacy of voice therapy, and it may help physicians to develop precise treatment for VFN patients (either by optimizing voice therapy or by performing surgery directly).

## 1. Introduction

Vocal fold nodules (VFNs) are mucosal lesions that occur in the anterior and middle thirds of the vocal folds [1]. VFNs are a significant challenge for otolaryngologists because VFN development is influenced by various medical, physiological, and psychological conditions; all variables must be investigated for diagnosis and treatment [2,3]. Treatments for VFN include medications, voice therapy, and microlaryngeal surgery. Voice therapy is useful for improving voice quality and reducing pathologic conditions [2,4]. Voice therapy strategies vary across clinicians and are dependent on patients’ individual needs and demands, motivation, and tissue characteristics [2]. Surgery can improve phonation and establish a pathological diagnosis of vocal fold lesion. Videostroboscopy is an effective tool for the initial evaluation of patients because of its speed and convenience [5]. Moreover, videostroboscopy with voice analysis before and after treatment has enabled clinicians to assess the dynamic movement of the vocal folds [6,7]. During videostroboscopy, the glottal area (GA) can also be recorded.

Glottal area waveform (GAW) analysis, presented by Timcke et al. in 1958, is a plot of relative GA versus time through a representative glottal cycle, and it can be used for the assessment of vocal fold vibration and function [8,9,10,11]. Conventional manual frame-by-frame GA analysis is time-consuming and is not a popular tool among clinicians [6]. Modern GAW analysis combines videostroboscopy and software, and it is quicker and more convenient than conventional GA analysis [12,13]. Montage images are created using videostroboscopy, and the maximum and minimum GAs are calculated using Image J software. High-speed videoendoscopy is an alternative assessment technique commonly used in the field of laryngology [14,15,16]. However, videostroboscopy has several clinical advantages. First, videostroboscopy can record long phonation samples. Second, data storage and retrieval procedures have been streamlined, providing immediate access to the recording for playback. Third, the real-time video can be played back with synchronous audio, which allows clinicians to refine clinical judgments [7,17,18].

The relationship between GA and treatment modalities in patients with VFNs remains unknown. The aim of this study was to investigate the maximum and minimum GAs of patients with VFNs before and after voice therapy or surgery based on videostroboscopy.

## 2. Methods

### 2.1. Patient Sources

We reviewed the medical records of 42 patients who had received treatment for VFNs from July 2015 to January 2017 at Chiayi Chang Gung Memorial Hospital. Patients with other concurrent laryngeal diseases such as laryngeal cancer, reflux laryngitis, vocal fold paralysis, vocal polyps, vocal cysts, and Reinke’s edema were excluded from the study. The enrolled patients were examined during their initial visit and followed up 3 months after voice therapy or surgery. Among these patients, 15 received voice therapy and 27 received surgery. For the voice therapy group, vocal function was recorded before and after treatment during at least three voice therapy sessions. For the surgery group, vocal functions were recorded 1–2 weeks before and 3 months after surgery. The study complied with the declaration of Helsinki. This study was approved by the Institutional Review Board of Chang Gung Memorial Hospital (No. 201800401B0).

### 2.2. Voice Analysis

Visibility of the entire vocal folds was required in videostroboscopy, and a complete cycle of /i/ phonation at a comfortable intensity and low frequency was recorded. Videostroboscopy was performed using a Kay Elemetrics Stroboscopy Unit (core model CSL 4500, KayPentax, Lincoln Park, Lincoln Park, NJ, USA). The acoustic parameters of average fundamental frequency (F0) in Hertz, shimmer, jitter, and noise–to-harmonic ratio (NHR) were recorded. Shimmer was expressed as the variability of the peak-to-peak amplitude in decibels, and jitter was the cycle-to-cycle variation in the fundamental frequency [19,20]. NHR is defined as the amount of additive noise in the voice signal used to evaluate a dysphonic voice [21]. Computerized Speech Laboratory (core model CSL 4500, KayPentax, Lincoln Park, NJ, USA) was used to measure acoustic parameters. Maximum phonation time (MPT) was defined as the maximum length of the vowel /i/ at a comfortable intensity [22]. The microphone-to-mouth distance was 5 cm. One laryngologist performed the endoscopy. A speech pathologist and an otolaryngologist analyzed the voice parameters in a double-blinded manner. If the patients were unable to complete the frequency tracking, they would be excluded in this study.

### 2.3. Subjective Assessment

The Voice Handicap Index (VHI) is one of the most psychometrically robust and well-studied instruments for measuring quality of life; the validities of the VHI-10 and Chinese VHI-10 (VHI-C10) have been demonstrated for distinguishing dysphonic and nondysphonic individuals as well as for documenting treatment effects among patients with dysphonia [23]. All patients enrolled in this study completed the VHI-C10 before and after treatment [23,24].

### 2.4. GA Measurement

**Figure 1:**
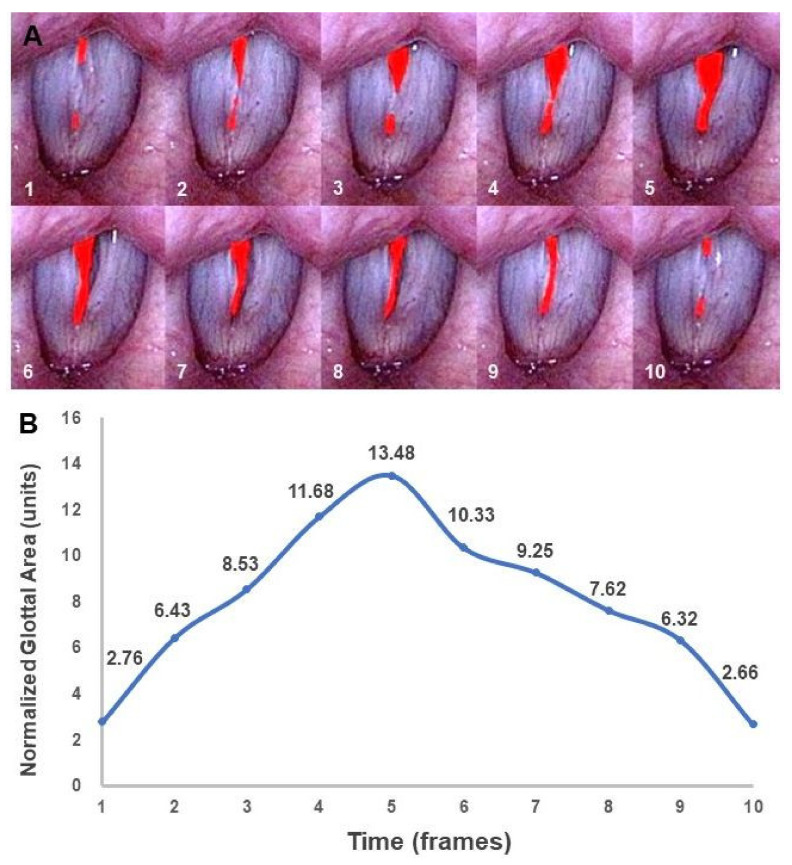
Glottal area for patients with vocal fold nodules. (**A**) A 10-frame sequence of the glottal area (the red-colored area). (**B**) Dynamic change of the normalized glottal area corresponding to the glottal area.

During videostroboscopy, approximately 10 serial images, each composed of one cycle of vocal fold vibration, were recorded. The maximum GA was measured at the beginning of the closing phase or the ending of the opening phase [10]. The minimum GA was measured at the beginning of the opening phase or the ending of the closing phase [10]. Maximum and minimum GAs were recorded from montage images. We selected images with maximum or minimum GA within each cycle from three vocal fold vibration cycles. The maximum and minimum GAs were calculated by computing the number of pixels composing the detected GA by using Image J, version 1.410 (NIH, Bethesda, MD, USA). Because GA is limited by the size of the patient’s larynx and the distance between the laryngoscope and the vocal folds, the comparison of GAs requires an internal reference for calibration. Hence, we used normalized GA, which was defined as the GA in pixels divided by the square of the glottal length (normalized glottal gap area (units) = glottal gap area (pixels × pixels)/(membranous vocal fold length)^2^ (pixels × pixels) × 100) [12,25,26]. Glottal length was defined as the distance between the anterior commissure and the vocal process. Figure 1a shows a representative example of a 10-frame GA sequence. We collected these montage images and observed the changes in normalized GA; we then calculated the maximum and minimum GAs (Figure 1b).

### 2.5. Statistical Analysis

The summary descriptive statistics are presented as means ± standard deviations for continuous variables. Changes in scores and data before and after treatment were analyzed using paired *t* tests using SPSS for Windows version 13.0 (IBM, Armonk, NY, USA); results with *p* < 0.05 were considered significant.

## 3. Results

### 3.1. Objective Voice Parameters

**Figure 2:**
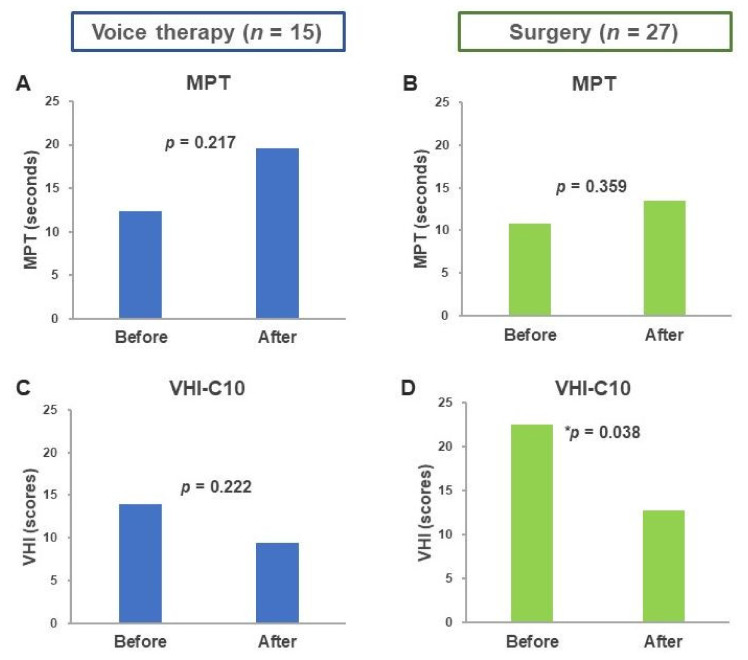
Differences in MPT and VHI-C10 before and after treatment. Changes in MPT before and after (**A**) voice therapy and (**B**) surgery. Changes in VHI before and after (**C**) voice therapy and (**D**) surgery. Abbreviations: MPT, maximum phonation time; VHI-C10, Chinese Voice Handicap Index-10, * *p* < 0.05.

The baseline characteristics and acoustic analysis results of the two groups are summarized in Table 1. No significant differences were observed in sex, age, and voice parameters (MPT, jitter, shimmer, and NHR) between the two groups; however, the VHI-C10 scores differed. The MPTs of the patients before and after voice therapy were 12.37 ± 5.47 and 19.55 ± 1.63, respectively (*p* = 0.217; Figure 2a). The MPTs of the patients before and after surgery were 10.77 ± 4.85 and 13.52 ± 8.99, respectively (*p* = 0.359; Figure 2b). The MPT improved following surgery and voice therapy, but the difference was not significant.

### 3.2. VHI-C10 Scores before and after Treatment

As shown in Figure 2c,d, the VHI-C10 score decreased nonsignificantly after voice therapy (14.0 ± 8.44 vs. 9.40 ± 10.24, *p* = 0.222) and decreased significantly after surgery (22.53 ± 7.17 vs. 12.75 ± 9.84, *p* = 0.038).

### 3.3. Maximum and Minimum Glottal Area

**Figure 3:**
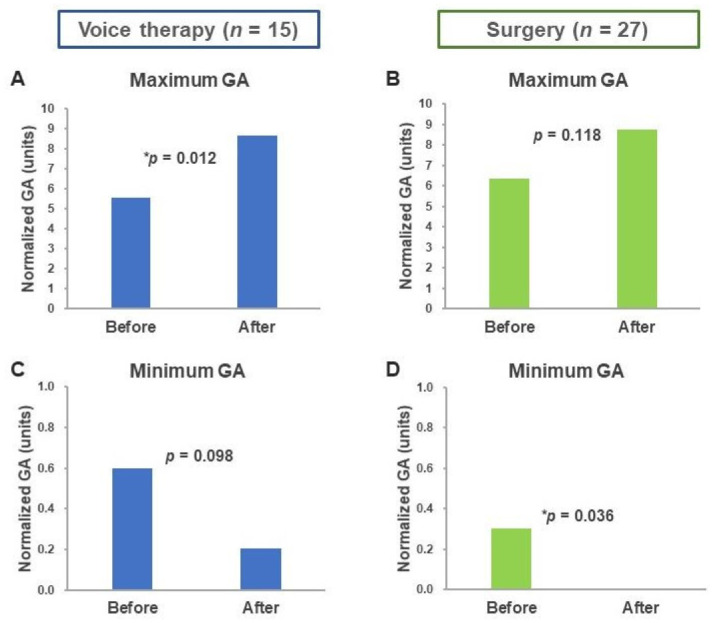
Changes in maximum and minimum GAs before and after treatment. Changes in maximum GA before and after (**A**) voice therapy and (**B**) surgery. Changes in minimum GA before and after (**C**) voice therapy and (**D**) surgery. Abbreviation: GA, glottal area. * *p* < 0.05.

The maximum GA of the voice therapy group was significantly increased after treatment (5.58 ± 2.41 vs. 8.65 ± 3.17, *p* = 0.012; Figure 3a). The maximum GA did not differ significantly after follow-up in the surgery group (6.34 ± 3.82 vs. 8.73 ± 5.57, *p* = 0.118; Figure 3b). By contrast, the minimum GA of the voice therapy group did not differ significantly between the groups before and after voice therapy (0.60 ± 0.73 vs. 0.21 ± 0.46, *p* = 0.098; Figure 3c), but it was significantly reduced after surgery (0.30 ± 0.59 vs. 0.00 ± 0.00, *p* = 0.036; Figure 3d).

## 4. Discussion

This study investigated the change in maximum and minimum GA in patients with VFNs before and after voice therapy or surgery. The results revealed that the change in GA was related to treatment modalities. Voice therapy significantly increased the maximum GA, and surgery significantly reduced the minimum GA. Through voice therapy, the power of closure was increased, the vibration pattern was optimized, and the maximum GA was significantly increased. However, the change in the minimum GA was limited after voice therapy because the nodule remained, especially in patients with large nodules. Because surgery eliminated the nodules, the minimum GA was reduced significantly.

Maximum and minimum GAs are associated with the efficiency of glottal closure [12]. Glottal closure is affected by many factors including vocal fold mucosal wave, mucosa lesions, and the tension of the vocalis muscle [26,27]. GA is associated with the airflow passage, and a larger GA is associated with a higher instantaneous volume velocity for a given level of transglottal pressure. When the airflow rate is higher, the subglottic pressure is lower. Thus, a patient’s voice is husky, and they are unable to sustain their voice for long. A recent study revealed that minimum GA is positively correlated to jitter and shimmer [28].

Voice therapy improved vocal function in patients with VFNs, indicating that it is an effective treatment for VFNs and should be considered as the first-line treatment. Surgery can also increase MPT and decrease VHI-C10 scores [29]. However, improper vocal fold surgery can cause vocal fold scarring. Permanent hoarse and breathy voice is caused by vibration impairment due to deterioration of the inner layers of the epithelium of the vocal folds. Through voice therapy, complications associated with surgery, including permanent damage to the vocal folds, can be avoided [30]. Voice therapy aims to change voice production patterns to minimize contact trauma during phonation. This treatment is the first-line treatment for VFNs because it typically resolves voice problems and prevents recurrence in most patients. Voice therapy usually involves education about etiological factors, vocal fold mechanics, and modification of specific vocal practices [2]. Although voice therapy can improve voice quality, complete resolution of VFNs is not always achieved. Surgery can then play a role in the treatment of VFNs.

The management of patients with persistent hoarseness after voice therapy is a key clinical challenge. However, no guidelines have been established to indicate whether laryngologists should continue voice therapy or to cease voice therapy and perform surgery. The results of this study enable physicians to better understand the mechanisms through which voice therapy and voice surgery improve voice performance. In brief, voice therapy significantly increased maximum GA and nonsignificantly reduced minimum GA. Surgery nonsignificantly increased the maximum GA and significantly reduced the minimum GA. Our findings could help physicians make clinical judgments for patients with persistent hoarseness after voice therapy. If stroboscopy reveals increased maximum GA and decreased minimum GA, then the voice therapy is effective but insufficient for the patient; therefore, we may suggest that the patient should receive surgery to improve their vocal performance. Conversely, if stroboscopy reveals no or limited change in maximum and minimum GA, the patient’s compliance with and quality of voice therapy should be carefully evaluated. In this situation, voice therapy can still be effective after the improvement of patient compliance, motivation, and training quality. Further prospective studies should be conducted to confirm this suggestion.

The present study had several limitations. First, the sample size was small, but it provided a meaningful finding regarding GA change in patients with VFNs. Second, although the software-assisted GA analysis method in this study was quicker and more convenient than conventional GA analysis, the selection of the frames to measure maximum and minimum GA still required personnel power. In the future, we believe that artificial intelligence could be applied for real-time GA measurement, enabling immediate evaluation of the effectiveness of voice therapy, surgery, or both.

## 5. Conclusions

This study investigated GA measurement in patients with VFNs following surgery or voice therapy. The findings indicated that voice therapy significantly increased maximum GA and surgery reduced minimum GA. On the basis of our study, GA analysis can be used to evaluate the effectiveness of voice therapy and may help physicians to formulate treatment plans for patients with persistent hoarseness after voice therapy (either by optimizing voice therapy or by performing surgery directly).

## Figures and Tables

**Table 1:** Baseline characteristics and acoustic analysis of enrolled patients.

Characteristics	Total Patients (*n* = 42)		*p* Value
Treatment	Voice therapy (*n* = 15)	Surgery (*n* = 27)	
Sex (Male/Female)	5/10	12/15	0.482
Mean age year (range)	52.28 (25–75)	48.0 (23–67)	0.324
MPT (sec)	12.37 ± 5.47	10.77 ± 4.85	0.666
Jitter	2.38 ± 2.16	3.10 ± 2.85	0.418
Shimmer	0.55 ± 0.41	0.58 ± 0.3	0.785
NHR	0.20 ± 0.14	0.18 ±0.09	0.624
VHI-C10	14.00 ± 8.44	22.53 ± 7.17	0.01 *

Abbreviations: MPT, maximum phonation time; NHR, noise-to-harmonic ratio; VHI-C10, Chinese Voice Handicap Index-10, * *p* < 0.05.

## Abbreviations

F0 = fundamental frequency, GA = glottal area, GAW = glottal area waveform, MPT = maximum phonation time, NHR = noise–to-harmonic ratio, VFN = vocal fold nodule, VHI = Voice Handicap Index, VHI-C10 = Chinese Voice Handicap Index-10.
